# Influencing Factors of Future Specialty Choice for Undergraduate Medical Students: An Updated Experience from the UAE

**DOI:** 10.1055/s-0043-1769931

**Published:** 2023-06-22

**Authors:** Abdulqader Al Zubaidi, Salama AlBuqaish, Alaa Ali, Mira Ibrahim, Shoroogh Marei, Shomous Nugud, Ahmed Nugud

**Affiliations:** 1 Department of Academic Affairs, Tawam Hospital, Al Ain, United Arab Emirates; 2 Department of Medical Affairs, Aljalila Children's Speciality Hospital, Dubai, United Arab Emirates; 3 Neurology Department, Rashid Hospital, Dubai Academic Health Corporation, Dubai, United Arab Emirates; 4 Department of Internal Medicine, Zayed Military Hospital, Abu Dhabi, United Arab Emirates; 5 College of Medicine, University of Sharjah, Sharjah, United Arab Emirates

**Keywords:** medical students, speciality preference, influencing factors, clerkship

## Abstract

**Background** Medical students' career choices determine the prospects of the future medical workforce, thus influencing the delivery of medical care. This study aims to identify and provide information about factors affecting the selection of future specialties among medical students.

**Methods** A cross-sectional study was conducted on students in both preclerkship and clerkship phases at a single institution in the United Arab Emirates. A self-administered questionnaire included questions about demographic data, most preferred specialties, and influential factors. The influential factors were measured using a Likert scale.

**Results** Surgery and internal medicine were the most desired specialties, respectively. Gender has a significant role in influencing career choice. There was no association between preclerkship and clerkship students' career choices. The most influential factors were seeing good treatment outcomes and having abilities for the specialty.

**Conclusions** Surgery and internal medicine were the most preferred specialties, even though significant gender differences existed in specialty choices among these students.

## Introduction

Medical education is an ongoing process that starts with medical school and progresses to specialization and subspecialization. Henceforth, in the early stages of medical education, medical students get exposed to a wide range of specialties. This exposure serves as the building block for students' future specialization direction. Despite this fact, even medical school applicants often have strong preferences for or against medical specialty. 1

These preferences have a significant impact on several important determinants of the healthcare services, 2 as medical graduates are a valuable part of the healthcare system, and they determine the medical workforce. 3

To date, factors that play a role in career preferences are partially understood, which can affect career choice before, during, and after medical school. 2 Factors that were identified in the previous studies showed that “medical school characteristics, personal interactions, and lifestyle preferences” are important factors that influence the students' choices. 4 Moreover, the clinical experience helps students to construct their professional identity and influence specialty choices in terms of views and approaches. 4 Therefore, understanding the student's views and directions by defining the factors that influence career choice is important to anticipate whether those views fit the expected demand for healthcare services and to use it in the process of policy implication to manage the number of specialties, especially in a current situation related to severe acute respiratory syndrome coronavirus 2 pandemic related shortage in qualified healthcare workers. 3 4 5

Health Authority of Abu Dhabi, United Arab Emirates (UAE), reported in 2013 an expected need of 2000 doctors and over 5000 nurses by 2022, which translates into 1500 doctors and over 2000 nurses to be recruited annually. Yet, these figures are not reflective of the new reality post-coronavirus disease 2019 pandemic. The main gaps in specialties like critical care medicine, emergency medicine, neonatology, pediatrics, oncology, and orthopaedics. 6

The matching system in the UAE follows a similar pattern to the matching system in North America, in which students must take a medical licensing exam “Emirates Medical Residency Entrance Examination (EMREE).” 6 The exam is usually taken by intern house officer physicians during their intern year. 2 After passing the exam, students can apply to the residency match under one of three governing bodies Department of Health that oversees the training in the emirate of Abu Dhabi, Dubai Academic Health Corporation that oversees the training in the emirate of Dubai, and Emirates Health Services that oversees the training in the norther emirates. 6 With most of the governing bodies awarding Arab Board of Health Specializations degree upon completion of training and successfully passing the required exams. 6 Overall due to limited training seats getting into medical residency is quite competitive in the UAE, with surgical specialities being more competitive. 6 The exact number of seats per specialty varies per year, but with an overall all estimate of around 59 seats in pediatrics, 55 seats in internal medicine, 39 seats in emergency medicine, and 17 seats in general surgery, with other specialities having a smaller number of seats. 6

A recent study in the UAE by Abdulrahman et al showed that students' career choices included internal medicine, surgery, and emergency medicine. Students reported factors such as intellectual satisfaction, work–life balance, secure future, and easier to find a resident position as the main reasons to choose a specialty. 5

A plethora of evidence pinpointed factors affecting medical students' career choices. Yet, unfortunately, there are not enough studies that explore the factors that affect the career preference for medical students in the region in general. Therefore, this study aimed to identify factors affecting the selection of future specialty among medical students. Such outcomes would help students and mentors utilize while choosing or enrolling in residency training programs in areas where such information is scarce. Also, outcomes would be of value to decision-makers given the expected rise in demand for medical specialists due to pandemic related stress on the health system and the increase in population number.

## Methods

### Study Population and Design

Data were collected using a cross-sectional study design. A self-administered questionnaire was distributed among medical students enrolled in years 1 to 5 at the College of Medicine during the academic year 2014 to 2015. The total number of medical students enrolled at our institution was 517 medical students; a minimum of 226 subjects was targeted to achieve statistical significance with a confidence interval (CI) of 95% and a margin of error of 5%. All the subjects were interviewed, questions were explained to all the students included in the study, anonymity was assured, and their participation was voluntary.

### Study Instrument

Data was collected through a self-administered questionnaire developed after a comprehensive review of relevant topics in the literature and using different questionnaires from previous studies. 2 7 8 9 The survey was developed in English by two experts in medical education and internally validated in a pilot survey of a randomly selected small group of students from the total student population of the college.

The questionnaire consisted of 43 questions divided into three sections. The first section determined the sociodemographic characteristics of the participant. In the second section, the participant had to choose the three most preferred specialties from 14 specialties which included: general medicine/family medicine, internal medicine subspecialties, surgery, pediatrics, obstetrics and gynecology, psychiatry, anesthesia, emergency medicine, dermatology, orthopaedic surgery, ophthalmology, otorhinolaryngology (ENT), urology, radiology, and basic science. In addition, a separate group of ''other'' specialties was constructed to include subjects like neurosurgery and forensic medicine. This list was chosen based on the popularity of listed specialties in clerkship and elective rotations. Certain subspecialties were not included in the study as the subspecialty choice is made after finishing the residency in major specialties. The third section provided 30 factors that the participant had to evaluate the degree to which the factors influenced their specialty choice on a 5-point Likert scale (1= Not at all influential, 2= slightly influential, 3= somewhat influential, 4= very influential, and 5= extremely influential); these factors covered the characteristics of specialty, the personal experience, the experience during clerkship, advice from others, and considering future work condition. The 5-point scale was collapsed to a 3-point scale (Not at all influential, Somewhat influential, Influential) during the analysis of the data to represent the percentage of students' responses graphically.

The questionnaire included two additional questions about how satisfied the students are with the opportunity provided during the medical college to explore potential career choice and about the need for career counseling in the process of choosing the specialty before starting the residency after completion of undergraduate studies. These two questions were added to assess the student's satisfaction with the medical curriculum to explore the future career choice and how much they are motivated to attend a session for career counseling for choosing the specialty.

The questionnaire included a consent form on the first page that the participants read and signed voluntarily without undue influence before answering any of the questions. The consent included the title of the study, brief information about the research, the name of the principal investigators, and their contact details. In addition, ethical approval from the University of Sharjah ethics committee was obtained prior to starting the study.

### Data Analyses

Data were entered into a database and analyzed using the Statistical Package for Social Sciences (SPSS) version 22. The 5-point Likert scale was collapsed into three categories (not at all influential, somewhat influential, and influential). After data entry, frequencies and percentages were computed. The chi-squared test was used to assess the association between specialty choices and the sociodemographic characteristics, academic characteristics, gender, and influential factors of the participants. It was also used to assess the significance of the factors in relation to the first chosen specialties individually. A two-tailed *p* -value of 0.05 is the cutoff value for significance. To ensure data confidentiality, data was saved on a password-protected external USB drive accessible only to the primary investigators.

## Results

The total response rate was 391 out of 517 students, around 75.6% of the total population agreed to participate. Females represented 61.4% of the respondents, while males represented 38.6% ( Supplementary Table S1 , available in the online version). Students at the preclerkship level represented 70.80%, and clinical clerkship turnout was 29.20%. The mean age of the study population was 20.7 ranging from 18 to 25 years old ( Supplementary Table S2 , available in the online version).

**Fig. 1:**
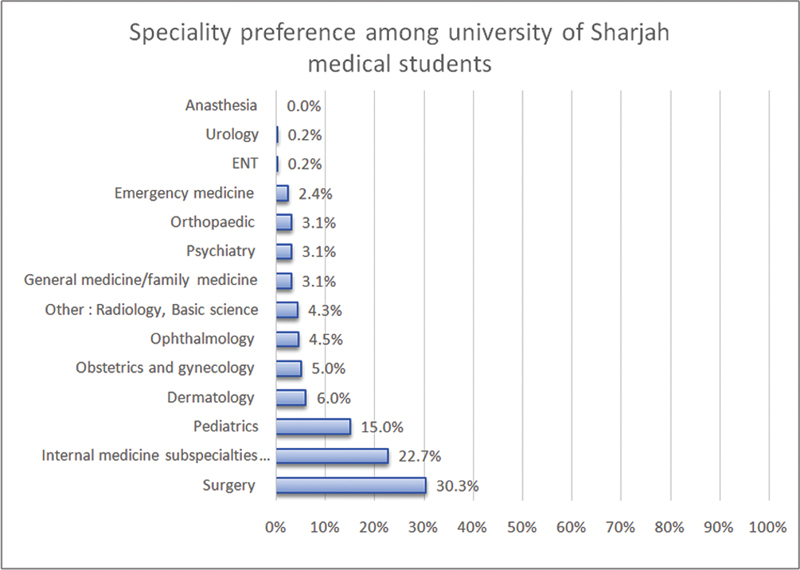
Speciality preference among medical students, *n* = 391.

The most preferred specialties included surgery 30.3%, internal medicine 22.7%, and pediatrics 15%. Meanwhile, ENT and urology were the least selected and only chosen by 0.2% as their preferred specialty. No one has selected anesthesia as a preferred specialty ( Fig. 1 ).

In our study, students who chose surgery were influenced by factors such as memorable experience at clinical rotation, followed by an interest in the surgical procedures and technologies, 78.8 and 77%, respectively. Another factor is a comfortable atmosphere at the specialty department (68.8%).

**Fig. 2:**
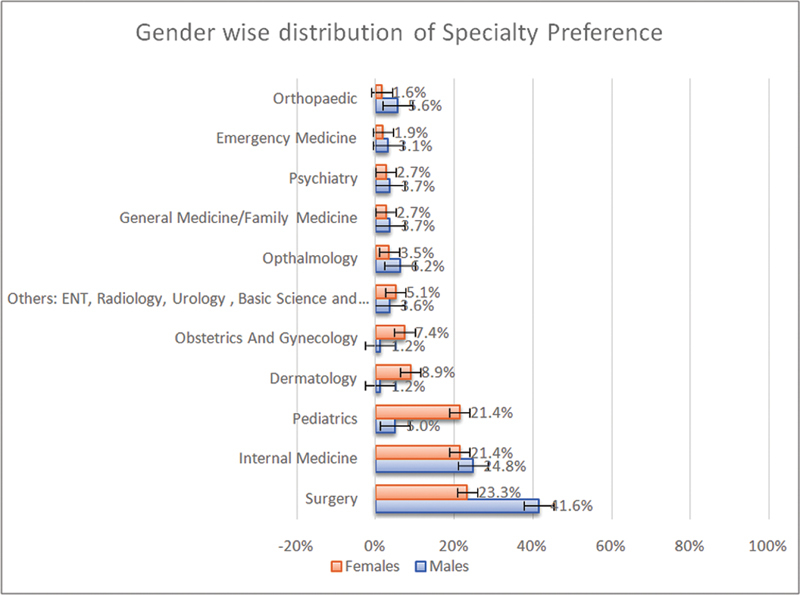
Medical student's future career preference by gender, *n* = 391.

On the one hand, the preferred specialties have a statistically significant difference between male and female medical students ( *p* -value < 0.0005); males were mainly interested in specialties like orthopaedics (69.2%) and ophthalmology (52.6%). While females showed interest in dermatology (92%) and pediatrics (87.3%) ( Table 1 ), the percentage of males who chose surgery is almost two times that of females; 41.6 and 23.3%, respectively. It is worth mentioning that internal medicine was chosen equally by both genders ( Fig. 2 ).

**Table 1:** Factors influencing specialty preference to have a significant difference between male and female students

Factors affecting the career choice	Male			Female			
	Not at all influential	Somewhat influential	Influential	Not at all influential	Somewhat influential	Influential	*p* -Value
Interest in the targeted population (e.g., children, the elderly)	32.50%	36.90%	30.60%	20%	29.40%	50.60%	>0.0005
Expected high income	25.60%	29.50%	44.90%	37.20%	31.20%	31.60%	0.013
Highly respected in society/good social life	15.60%	26.30%	58.10%	26.50%	24.90%	48.60%	0.031
Not interested to have a direct interaction with patients (in following up)	48.70%	25.90%	25.30%	62.50%	20.20%	17.40%	0.022

**Fig. 3:**
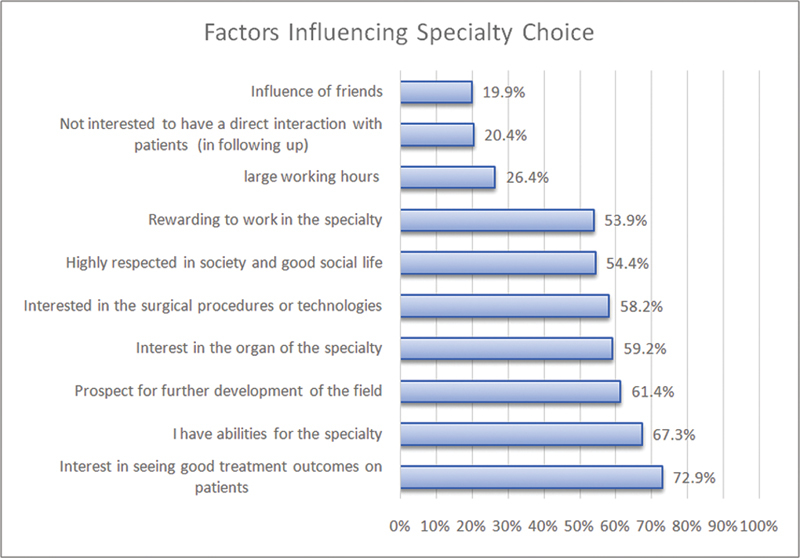
Factors influencing specialty preferences among medical students, *n* = 391.

On the other hand, there was no association between preclerkship and clerkship students' career choices ( *p* -value = 0.226). Association between year allocation, whether basic medical sciences (years 1-3) or clinical sciences (years 4-5), seems to impact students' preferences ( *p* -value = 0.014). Students reported that experiencing satisfactory patient treatment outcomes in the specialty would affect their choice 72.9% of the time. Students who chose surgery were influenced by factors such as memorable experiences at clinical rotation, followed by an interest in surgical procedures and technologies, 78.8 and 77%, respectively. Another factor is the comfortable atmosphere in the specialty department (68.8%). Other factors, such as the perceived skills required for a particular specialty (67.3%) and the prospect for further field development (61.4%), were also considered during decision-making. Other factors reported to have some influence included recommendations from friends and family influenced career choice in 19.9%, loss of interest to have direct interaction with patients in 20.4%, and the span of working hours in 26.4% ( Fig. 3 ).

Generally, both genders leaned towards specialties with higher income rates and viewed it as an influential factor while choosing their training with a CI of 40.15 to 49.65 for males and 27.16 to 36.04 for females, and the *p* -value was 0.013. Meanwhile, the targeted population who would receive medical services also played a major role during the training selection process, particularly for females with a 95% CI of 45.82 to 55.38, whereas a lesser degree of males 95% CI of 26.2 to 35 viewed the end-user served population would affect their specialty selection, *p* > 0.0005. On the same note, both genders cited lesser interest regarding direct interactions or follow-ups with patients post services, with a *p* -value of 0.022. In addition, perceived views about specialties in terms of respect and social acceptance, and work/life balance played a lesser role while the specialty choosing process with approximately 25.6%, *p* -value of 0.031.

Role models influenced 46% of the students included in our study. Moreover, 91.6% of the students were interested in attending a specialty counseling workshop before starting the residency and after completing undergraduate studies, while 8.4% were not interested in such an activity. Furthermore, students' satisfaction with opportunities for guidance toward potential career choices during undergraduate medical studies and training showed that 22.8% were satisfied, and 47.7 and 29.5% were neutral and dissatisfied, respectively.

## Discussion

**Fig. 4:**
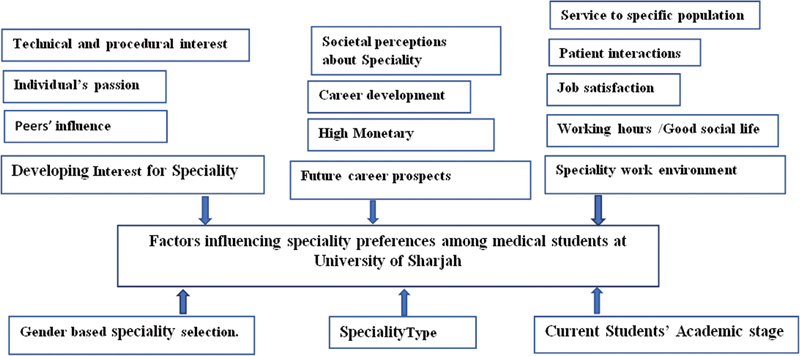
Thematic map of factors influencing specialty preferences among medical students.

Factors influencing a medical students' future career choice vary with culture, personal experience, and progression through the medical school curriculum. Knowledge of such factors aid decision-makers and other stakeholders in planning for a given country's future medical workforce. Fig. 4 shows a thematic map of the identified factors. With limited training spots and an increasing number of medical graduates, hence increasing competitiveness of the selection process, factors that affect medical student's career choice play a pivotal role in the future of any healthcare system. With a recent decree in the UAE that application to training is only allowed up to 4 years after graduation from medical school, medical students' specialty choice is time critical. This study showed a similar trend to other gulf countries regarding medical students' career preference, as the most preferred specialties were surgery, internal medicine, and paediatrics. 8

Previous reports showed that the vast majority of students showed interest in internal medicine, surgery, emergency medicine and critical care, and family medicine as their specialty of choice. 7 8 10 11 12 A similar trend was seen globally in resource-rich and resource-poor countries, including KSA, Kuwait, Japan, Canada, Greece, Sudan, and Jordan. 7 8 10 11 12 Henceforth, medical students in the UAE show similar career preferences to their peers in other parts of the world. This can be attributed to students' exposure during clinical and preclinical years in these specialties.

Our results advocate similar findings of previous studies done in Jordan and Sudan. 10 11 In the gulf region, surgery was reported as the most preferred future specialty by medical students, similar to our results, as surgery was also the most preferred specialty within both genders. 1 2 8 Furthermore, worldwide surgery was listed in the top three selected specialties. 4 7 12 This finding can be explained by the opportunity to perform practical procedures and operations during clerkship in surgical versus nonsurgical specialties.

The above can be further explained via other factors. In our study, students who chose surgery were influenced by factors such as memorable experiences at clinical rotation, followed by an interest in surgical procedures and technologies. These factors show that the opportunity to perform practical procedures and operations is an important factor that makes students like surgery a future career choice. Another factor is the comfortable atmosphere in the specialty department. This all adds up to the principle of exposure, which indicates that exposure to the medical field can have a role in building interest in the specialty.

Furthermore, role models can affect and impact students' choices, whether to choose a specialty or to drive away from a particular specialty. The role model can be a parent or other relative, personal physician, family friend, or even a medical school faculty member. In most studies, relatives' or seniors' advice played a major role in the specialty choice. 2 11 12 However, this was not seen in our study. Students instead were influenced by treatment outcomes expectations in the specialty itself (e.g., critical care medicine compared to pediatrics), specialty compatibility, and field development. However, Alawad et al, in 2015, reported that 46% ( *n* = 887) of their cohort had role models from their families as an impactful reason for their preferred specialty choice and information. 10 Other than this, memorable experiences during clerkship rotations influenced students' career preferences, which indicate that exposure to the medical field can have a role in building an interest in the speciality. 10

Anesthesia was the least selected specialty, which is consistent with other studies done in the Kingdom of Saudi Arabia. This can be explained by students' minimal exposure to anesthesiology, which in turn is attributed to the low weight in students' curriculum compared to other specialties. 1 Another reason could be a lack of awareness about the clinical responsibilities the specialty encompasses and possible underrepresentation in social media and TV shows. 2

An association between gender and specialty choice was found in our study. Khader et al, in 2008, found that the most preferred specialty among male students was surgery, followed by internal medicine and orthopaedics, while the most preferred by female students was obstetrics and gynecology, followed by pediatrics and surgery. 11 However, in our study surgery was the most preferred specialty by both genders. The percentage of males is almost two times that of females, while females were five times more likely to choose pediatrics than males; these findings align with Alawad et al findings from Sudan. 10

An interesting observation by Zolaly et al, in 2013, reported marital status for females as a factor in future specialty preference, while this was not a factor in their male counterparts, which reported work achievement-derived career choices in males. 13

The gender ratio seems to have contributed to our final results as the majority of respondents were females, despite the fact that pediatrics was chosen as one of the most preferred specialties overall. Similarly, this was seen in the study conducted in Kuwait, where their gender ratio was (57.1%: 42.9%), and pediatrics was their first preferred specialty. 8 Bittaye et al, in 2012, findings showed that career preference might vary according to gender, similar to our study findings. In contrast, factors such as residency program duration and lack of subspecialists in the field of interest had the same impact. On one hand in our study, expected income and targeted population under services (children, elderly, males or females, for example) had a significant impact on students' decision during the selection process of a preferred specialty, on the other hand Bittaye et al 2012 findings did not show a similar trend.

Although there was no follow-up for the student during their medical years, the results showed that the specialty preferences differed across years of study. Yet, based on Abdulrahman et al, 5 findings can help elucidate a trend in the country. Our result showed a trend to select surgery in the first, second, and fifth year, while internal medicine in the third and fourth years. This finding agrees with the fact that exposure to different fields through undergraduate medical studies changes the students' perception of the specialties and develops within the students some feeling for or against different specialties, which was found in previous studies. 11 12 14 Yet it is worth mentioning that a significant association was found between progress through medical school (basic sciences phase compared to clinical sciences), *p* -value = 0.014.

The additional questions responses showed the students' interest in attending a workshop on career counseling before starting the residency and after completion of their undergraduate studies. This reflects the need to provide support to the students, which will help them to determine the best way for them in the residency program and help to distribute the future students with regard to their abilities and interest in the fields that have capacity gaps. This is compatible with what was found in the previous studies. Therefore, we recommend the establishment of a career counseling department in the college and providing sessions of career counseling during medical education. 2 7 15

### Limitations and Future Plans

The limitations of the study are as follows: first, the study was conducted in one institution and would not fully reflect the national trend, yet this was compensated for as there was a previous study done locally a few years earlier and was used as a baseline to compare for trends changes in specialty preferences among medical students and an indirect way to follow up students. In addition, the study tool was only internally validated via a pilot study at our institution. Furthermore, there were few double-barreled questions in the survey that might obscure the respondents' true intentions. Our study did not investigate medical students' summer electives as a factor in specialty selection, which can be done in future studies. The results might have been skewed by the number of nonrespondents despite a good response rate; this could have been further investigated by sensitivity analysis of respondents versus nonrespondents. We also recommend career counseling not only to senior students but rather across the board to all medical students in a periodic manner, as well as prospects of research in the fields of interest as a factor in future specialty decisions. In addition, doing a prospective cohort study to look at the same group of students' interests and factors that might affect their specialty choice can give another perspective on confounding factors.

## Conclusion

Surgery and internal medicine were preferred specialties by both genders. Significant gender differences existed in specialty choices among students that were attributed to factors like future prospects, income, served population, treatment outcomes, and possessing the right abilities needed for training. Lack of exposure and knowledge of certain specialties were among the reasons for the lack of interest in some specialties. Career counseling and elective training in specialties of interest can help medical students ascertain their perspective desired specialties.
