# Supplementary material for: Influencing Factors of Future Specialty Choice for Undergraduate Medical Students: An Updated Experience from the UAE
Source: Avicenna J Med. 2023 Jun 22;13(2):97–103. doi: 10.1055/s-0043-1769931 (PMC10332941; doi:10.1055/s-0043-1769931)
Supplement: Supplementary file 1 — Supplementary Material [file 10-1055-s-0043-1769931-s210104.pdf]

Supplementary Table S1 Gender percentage of study respondents per year of study, $n = 391$

Year of enrolment	Males	Females
Year 1	27.2%	25.2%
Year 2	21.0%	27.5%
Year 3	21.6%	18.6%
Year 4	16.7%	16.7%
Year 5	13.6%	12.0%

Supplementary Table S2 Age distribution of study respondents, $n = 391$

Age (years)	Percentage
18	3.3%
19	21.5%
20	23.8%
21	22.5%
22	17.1%
23	7.4%
24	3.1%
25	1.3%

 © 2023. The Author(s).
